# Nemone Lethbridge’s play *Baby Blues* on BBC television: maternal mental illness narratives, stigma and support in 1970s Britain

**DOI:** 10.1080/09612025.2024.2327895

**Published:** 2024-03-15

**Authors:** Fabiola Creed

**Affiliations:** Centre for History of Medicine, University of Warwick, Coventry, UK

**Keywords:** Postnatal depression, postpartum psychosis, *Baby Blues*, infertility, stigma, women’s narratives, Nemone Lethbridge, British Broadcasting Corporation (BBC), grassroots organisations, Depressives Anonymous (DA)

## Abstract

In December 1973, the BBC aired Nemone Lethbridge’s auto-fictional play *Baby Blues* as one of their influential ‘Play for Today’ (PfT) series (1970–1984). This article explores the impact of Lethbridge’s controversial television play, which drew attention to taboo topics, such as infertility, caesarean section childbirth, infanticide, suicide, and, separately, motherhood ageism and dismissive medical professionals. It will illustrate how Lethbridge’s play *Baby Blues* was part of a broader change in discussing maternal mental illness and creating support for women experiencing postnatal depression and psychosis, instigated by the Women’s Liberation Movement (WLM). The article situates *Baby Blues* within the wider history of the PfT series, with its focus on socio-political issues, and highlights the challenges Lethbridge faced in bringing the play to production. It analyses the mixed responses to the play, many of which were critical, and how this led to Lethbridge’s launching of a grass-roots self-help group, Depressives Anonymous (DA), in 1974, which was—and still is—a long-lasting legacy of *Baby Blues*. The article builds on the history of maternal mental illness as explored in women’s narratives and its association with stigma, support and feminism, alongside the British Broadcasting Corporation’s television series PfT, in 1970s Britain.

## Introduction

Before the 1970s, women of all social backgrounds were afraid to publicly disclose that they had depression, never mind puerperal depression. This article explores how Nemone Lethbridge’s television Play for Today (PfT), *Baby Blues* (BBC1, 1973), helped change this. The play narrates the story of a woman in her late thirties who, after struggling to conceive for ten years, experiences postnatal depression and psychosis after the delivery of her first baby by caesarean section. She then catches her husband sexually cheating with their young au pair. Her mental health deteriorates even further, and she makes the decision to kill herself and her ‘deeply loved’ child to stop both their suffering.^1^ Lethbridge’s experience of maternal mental illness formed the basis of this auto-fictional play, which caused controversy at the time of its release. Yet, Lethbridge and her play contributed to the de-stigmatisation of mental health in Britain by prompting discussions on maternal mental illness before such conversations were widely acceptable and by creating a support network for suffering mothers.^2^ This article, therefore, deepens the history of maternal mental illness as explored in women’s narratives and how the women sharing their experiences influenced both mental health stigma and support in 1970s Britain.

By offering a new perspective to the scholarship on PfT’s women writers, it also extends the history of the British Broadcasting Corporation’s (BBC) television series PfT (1970–1984) in early 1970s Britain.^3^ PfT was one of the first series pioneered by predominantly working-class people who were employed by the BBC and wanted to deploy ‘ordinary’ experiences to address socio-political issues. *Baby Blues* explored infertility, ageist attitudes in relation to motherhood, caesarean section births, women’s mental health and dismissive medical professionals, all controversial and little-explored issues.

Lethbridge’s own suffering, which inspired the play, was experienced during a period when medical professionals could not agree on how pregnancy and birth affected women’s mental health. While doctors and the media were becoming more interested in maternal mental illness in the 1970s, more commonly using the terms ‘puerperal’, ‘postpartum’ and ‘postnatal’ depression or psychosis, both illnesses were often unrecognised. It was also unusual for medics to diagnose mothers with both depression and the more severe but rarer condition of psychosis.^4^ Lethbridge herself was diagnosed with both depression and psychosis in July 1971 after suffering for eighteen months.^5^ As doctors and the media interchangeably deployed these labels to depict Lethbridge’s illness, I will use them alongside the broader term maternal mental illness.

To illustrate how Lethbridge’s play was part of a broader movement that supported greater awareness and understanding of maternal mental illness, this article first highlights the number of accessible sufferers’ testimonies and support networks before and after *Baby Blues*. Before *Baby Blues*, mothers lacked a safe media space to share their experiences of maternal mental illness, and there was little grass-roots support. Socio-cultural historian George Stevenson argues that, in Britain, the Women’s Liberation Movement (WLM) emerged in 1968.^6^ This was the year before Lethbridge became pregnant. Although decades apart, both feminist historian Sheila Rowbotham and television scholar Vicky Ball argue that Lethbridge’s play was an example of WLM’s consciousness-raising media. Like other feminists in support of the WLM, Lethbridge deployed *Baby Blues* to challenge institutional sexism and campaign for women’s political, economic, social and, therefore, domestic equality.^7^ Inspired by the WLM, Lethbridge tackled the taboo associated with maternal mental illness by opening conversations on the subject and advocating the creation of support networks. In doing so, Lethbridge used her position of privilege to raise awareness of women’s issues to support feminism.

By mapping Lethbridge’s background and life events from the 1950s and 1960s, this article will illustrate how Lethbridge protested against institutional gender and class inequalities and raised awareness of women’s other health issues, such as infertility, before the official WLM. This section will also demonstrate how Lethbridge’s background influenced her writing of *Baby Blues*. I will then trace the class and gender dynamics of the BBC as an employer and broadcaster at the start of the PfT series, which will contextualise the resistance Lethbridge faced when trying to commission *Baby Blues*.

Next, an analysis of *Baby Blues* will show how Lethbridge used her experience to exemplify the challenges of childbirth and mental illness. These included ‘high-risk’ pregnancies, the roles of medical professionals, the medicalised hospital experience and the rituals surrounding childbirth. The article will then trace the mixed reception from critics and viewers in the print press and the BBC's Audience Research Report. The controversial reception gave Lethbridge major publicity and recognition, which influenced feminists and journalists to position Lethbridge as a critical figure on the subject matter. Lethbridge soon became recognised as a person who relentlessly supported women with maternal mental illness despite media pushback. Lethbridge consequently launched a grass-roots self-help group, Depressives Anonymous (DA), in 1974. I conclude by examining the rise of DA, which was—and still is—a long-lasting legacy of *Baby Blues*.

This article applies a feminist approach by aiming to understand the nature of gender inequality from the perspectives of women. Consequently, I will illustrate how the growth of the WLM, the PfT series and grassroots mental health support groups intertwined by examining sources pertaining to Lethbridge, her play and DA. To highlight how Lethbridge used her lived experience to write *Baby Blues*, this article draws on Lethbridge’s play script, the televised play itself, her letters to several BBC producers and directors (BBC Written Archives), and autobiographical books by Lethbridge and her children’s father, Jimmy O’Connor. These include O’Connor’ *The Eleventh Commandment* (1976), Lethbridge’s *Nemone* (2021), and her ‘infertility’ chapter in a feminist book, *Woman on Woman* (1971).^8^ Even though Lethbridge accessed privileged medical and childcare support, oral histories from the British Library illustrate the similarities between Lethbridge’s and other women’s maternal mental illness experiences in the 1970s.

Moreover, the article explores how several journalists, and often Lethbridge herself, discussed *Baby Blues* in specialist political magazines (*New Statesman)*, feminist magazines (*Spare Rib*) and radio, theatre and television press and programmes (*Radio Times*, *The Stage*, *Television Today* and *Woman’s Hour*). I also evaluate how Lethbridge, journalists, and tele-viewers through their letters, both addressed and reacted to her play in a number of regional and national newspapers.^9^ Although the specialist magazines were perhaps restricted to middle-class readers interested in the arts, politics and feminism, the vast array of regional and national coverage on *Baby Blues*, and the *Woman’s Hour* radio programme in particular, had the potential to reach millions of people of all genders, ages, and socio-economic backgrounds. The play alone featured on Britain's most widely watched television channel, BBC1, and according to viewing figures, *Baby Blues* reached 7.3 million people when it aired on 6 December 1973.^10^

To situate Lethbridge’s play within debates on and approaches towards women’s mental health narratives in both media and political contexts, I draw on one of psychologist Jane Ussher’s theories on maternal mental illness. Ussher argues that women’s mental illness, in general, is a ‘reasonable response’ in the context of women’s lives and not necessarily a pathology from within. She explains how postnatal depression, in particular, is an ‘understandable’ and ‘normal’ response to one of the biggest changes and ‘greatest stressors’ in family life. Ussher explains how the diagnosis pathologises women who are tired, unhappy, and overwrought following childbirth and early child-rearing. Ussher further suggests that the diagnosis maintains that women need a biomedical cure, in trend with the medicalisation of childbirth in the twentieth century.^11^ Although Ussher's argument appears reasonable, it risks both supporting a ‘one size fits all’ approach and delegitimising some women’s mental illness.

However, Ussher highlights the importance of historicising women’s mental illness narrations because it grants women social viability, visibility and, therefore, support.^12^ As Lethbridge’s play will illustrate, unravelling women’s mental health also ‘provides insights into the gendered nature of social and familial life, the consequence of inequality and discrimination for both women and men’, and the cultural differences in how women process mental illness.^13^ Lethbridge’s personal experience and writing of her auto-fiction play, therefore, offers insights into how postnatal depression and psychosis connected to feminism, medicine, culture and the media in the 1970s. Moreover, it will demonstrate how Lethbridge, as a feminist activist, both participated in and challenged the medical and cultural policing of mothers’ bodies.^14^ Collectively, this article on Nemone Lethbridge and her play *Baby Blues* contributes to broader scholarship on gender, medicine, television and culture by deepening the limited historiography on maternal mental illness narratives.

## Maternal mental illness narratives, stigma, support and feminism in early 1970s Britain

Lethbridge and her play were deeply embedded in the feminist movement of the time and, therefore, part of a broader focus on trying to improve maternal mental illness support in Britain. As historian of motherhood Angela Davis argues, people were already focused on improving women’s childbirth experiences, alongside the standards of maternity care, after World War Two and the National Health Service (NHS) launch in 1948, when childbirth services became free for everyone.^15^ However, women were said to be experiencing ‘inhumane’ conditions following the shift in birthplace from the home to the hospital, which rose from 68.2 to 91.4 per cent between 1963 and 1972.^16^

As historian Sue Bruley explains, these changes occurred at a time of significant campaigning for improved reproductive rights, education, and work opportunities for women, contributing to the birth of the WLM in the late 1960s.^17^ The WLM and Lethbridge herself understood that women wanted their voices to be heard in medical and media spaces to improve women’s experiences of health and childbirth. By the early 1970s, feminists were routinely scrutinising medicalised childbirth practices and maternity care provision, as Lethbridge did in her play, yet maternal mental health was less explored.^18^ Some medical professionals, like psychiatrist Brice Pitt, were acutely aware of the impact of maternal mental illness, but many doctors and most feminists were not.^19^ As such, Lethbridge, a left-wing feminist, helped place maternal mental illness at the forefront of public, feminist and medical considerations.

When Lethbridge wrote her auto-fictional script, fought for its production, and had her televised play reach millions of viewers on BBC1, according to Ball, public discussions on maternal mental illness were taboo.^20^ Lethbridge’s play initially exacerbated this stigma. For example, in interviews, Lethbridge spoke about her experience of postpartum psychosis and openly shared that the worst outcome—an escape through infanticide and suicide—had been on her mind throughout her suffering.^21^ Nonetheless, as Ball notes, *Baby Blues* placed a crucial spotlight on the mental health politics of motherhood in Britain. Lethbridge’s inclusion of infanticide challenged and complicated the ‘normative’ maternal bond and the ‘self-sacrific[ing]’ mother.^22^

Before *Baby Blues*, national newspapers, women’s magazines, radio, television, and film had not yet discussed maternal mental illness experiences from a mother’s perspective. After the airing of *Baby Blues*, Lethbridge spoke about maternal mental illness in all these mediums, which, in turn, created an opening for other women to share their experiences and support for the first time mutually. For example, the feminist magazine *Spare Rib*, which was established in 1972, began to address postnatal depression shortly after the publication of Lethbridge’s ‘Postscript to *Baby Blues*’ article in 1974.^23^ According to gender historians Lucy Delap and Zoe Strimpel, *Spare Rib* was ‘Britain’s most high-profile feminist magazine’ during the WLM.^24^ Known as ‘one of the most iconic and long-lasting achievements’ of Britain’s WLM, the monthly magazine spanned the movement’s most active decades, discontinuing in 1994. *Spare Rib* encouraged women to speak up about controversial issues.^25^ As such, the magazine invited and then published Lethbridge’s first in-depth article on *Baby Blues* and featured Lethbridge on their front cover in 1974.^26^ By 1987, fifteen years and 180 issues since *Spare Rib*’s initiation, Lethbridge’s article on postnatal depression had still stimulated *Spare Rib*’s largest reader response.^27^ The article made maternal mental illness a common discussion point in *Spare Rib* until the magazine went out of print.^28^

Moreover, the BBC’s renowned *Woman’s Hour*, first broadcast in 1946, only addressed puerperal depression from a woman’s perspective in 1974, despite being a programme largely dedicated to topics related to motherhood. This report and personal testimony on *Woman's Hour* was prompted by *Baby Blues* and included an interview with Lethbridge.^29^ *Baby Blues* likely contributed to changes in television too, as documentaries (*Having a* Baby, BBC2, 1977) and auto-fictional depictions (*No Mama* No, ITV, 1979) on the personal experiences of maternal mental illness became standard by the end of the decade.

As Sarah Crook has illustrated, the few medical groups and organisations interested in developing better networks of expertise and support for people with maternal mental illness tended to apply a top-down, drawing on medical expertise rather than a grass-roots approach to campaigns and outreach before the 1970s.^30^ Many doctors were reluctant to acknowledge the severity of maternal mental illness and they often disregarded mothers until they harmed themselves or their baby(s). When mothers did ask for support from doctors, those who sympathised and acknowledged the illness typically sent women home with anti-depressants or sedatives.^31^ Lethbridge’s ‘radical’ play and the watershed in media responses that it triggered were reflective of the wider WLM; it all prompted taboo discussions on maternal mental illness with the aim to increase women’s awareness and support.

## Nemone Lethbridge

Lethbridge was a ‘fierce’ feminist before the WLM, and her background helped her overcome many setbacks, including those related to the commissioning and broadcasting of *Baby Blues*.^32^ In 1932, Lethbridge was born into an aristocratic family.^33^ Throughout her youth, she was engaged in politics and had a strong sense of justice. After boarding school, Lethbridge became one of only two women applying to study law at the University of Oxford in 1952. As the women-only Somerville College did not have a law tutor, they were sent to Keeble College, where their male tutor mocked the idea of women barristers. Nevertheless, by the mid-1950s, Lethbridge became one of Britain’s first female barristers, specialising in criminal law, despite much resistance. When barred from facilities, cases and even briefings, Lethbridge resourcefully developed ways ‘to find [her] own work’.^34^ She later reflected this same resilience throughout the commissioning and reception of *Baby Blues*.

From the outset, Lethbridge acknowledged how her privileged background enabled opportunities out of reach for most women and was empathetic to people without ‘nepotism’ and aristocratic advantage.^35^ Lethbridge recognised that she could more easily change women’s norms and expectations regarding education, career, and later motherhood. Moreover, similar to her feminist inspirations—Rose Heilbron, Jean Southworth, and Elizabeth Ann Curnow, who were some of the first women barristers and later judges in England—Lethbridge supported other women throughout their life successes and failures when it was not typical to do so.^36^ Lethbridge’s male-orientated and competitive career choice—where it was easier to slander the women pitted against her—supports this. Additionally, Lethbridge did not discriminate along the lines of class. This later informed her decision-making behind *Baby Blues*, her marriage, and the creation of her self-help group.

Unlike Lethbridge’s high society parents, Lethbridge’s future husband, Jimmy O’Connor, came from a background of poverty. O’Connor survived on London’s streets as a petty thief, moving in and out of prison. In 1942, he was falsely convicted and sentenced to death for murder, but an intervention led to O’Connor serving an eleven-year life sentence instead. When released in 1952, O’Connor became a crime reporter for ten years, but his dream was to write plays based on his personal experiences.^37^ In 1958, O’Connor met Lethbridge in London. Despite O’Connor being fourteen years her senior, they bonded over their love of writing and interest in crime. Lethbridge knew society would reject their relationship because of their polarised backgrounds.^38^ Nonetheless, in 1959, they married in Dublin, Ireland, to stop English journalists from finding their marriage records. However, the *Telegraph* exposed their relationship years later, in 1964, and the Bar asked Lethbridge to resign. Lethbridge said that she was asked to leave because, as a ‘left-wing woman’, she could not both practice law and be married to a man with a conviction.^39^ Nonetheless, she adapted; Lethbridge and O’Connor sold their ‘Beauty and the Beast’ story to the newspapers to build a home and live on the Greek Island of Mykonos.^40^ Lethbridge then focused on conceiving a baby.^41^

## (In)fertility

Lethbridge’s experience and her play offer valuable insights into the history of both (in)fertility and women’s ageism in the 1960s, and the differences between public and private healthcare. Her decade-long struggle started when the contraceptive pill was introduced for married women in the early 1960s and for all other women regardless of marital status or age by 1974.^42^ Driven by the WLM, conversations on reproductive control were becoming more common; however, cultural pressures concerning the ‘right’ type of women—not ‘disreputable’ or high-risk ‘elderly’ women—to bear children had not lessened.^43^

Lethbridge’s medical and cultural experience of (in)fertility contributed to her development of maternal mental illness and the experiences she shared in *Baby Blues.* From the outset, Lethbridge was ‘very family-minded’ and desperately wanted children after marriage.^44^ After four years of marriage and trying to conceive, Lethbridge visited a National Health Service (NHS) doctor. The doctor sent her to the Fertility Clinic or, as she described it, the ‘Futility Clinic’. As the waiting list for a hysterosalpingogram operation (a procedure to inspect the inside of the uterus and fallopian tubes for irregularities and blockages) was many months long, Lethbridge visited a private ward. The tests confirmed that ‘there was nothing wrong with [Lethbridge] or [her] husband’. After five years of trying, the doctor impatiently instructed Lethbridge to ‘stop fussing’, not to return, not to be conned by ‘clever gynaecologist[s]’ and that nothing else could be done. Typical of Lethbridge, she ignored her private doctors and spent another three years visiting Harley Street Hospital and the Samaritan Hospital. Lethbridge was determined and endured more tests, another hysterosalpingogram operation, several curettages (a procedure where the cervical canal and uterine lining are scraped to remove abnormal tissues), and a laparotomy (a surgical incision into the abdominal cavity to examine the abdominal organs for any problems). She also tried adoption; however, the adoption services rejected Lethbridge because of her ‘scandalous reputation’ and O’Connor’s criminal record.^45^

Regarding her experience, Lethbridge explained that the ‘infertile woman exists in a kind of limbo’ in the *Guardian* newspaper. She felt anxious when surrounded by babies, and some mothers hurtfully considered Lethbridge a ‘baby-snatcher’. Although Lethbridge and O’Connor tried to focus on other aspects of their ‘blissful’ new life in Greece, the ‘baby-crazy’ Greeks had no hesitations in asking why Lethbridge was ‘childless’. Lethbridge even experimented with a Japanese infertility treatment involving agonising Vitamin C and hormone injections, and, for the first time, she reached eight weeks of pregnancy. When Lethbridge miscarried, the couple felt a more profound sense of loss. Lethbridge returned to Harley Street Hospital after eleven years of trying to conceive. Aged thirty-seven years, the doctors commented on her age and the ‘typical picture of declining fertility’, leaving her in tears. However, three months later, in June 1969, Lethbridge fell pregnant and reached full term.^46^

As Lethbridge was significantly affected by her infertility, she wrote about her experience when Margaret Laing invited her to write a chapter for Laing’s edited collection *Woman on Woman* (1971). Laing was a feminist, a WLM supporter, and a leading writer and journalist who was once the editor-in-chief of the *Woman’s Mirror*.^47^ In Lethbridge’s essay, she explained how infertility was ‘demoralizing’ because she had ‘failed’ in the ‘world’ of women.^48^ In Gillian Tindall’s review of *Woman on Woman* in the *Guardian*, Tindall described Lethbridge’s chapter as a ‘modest and personal account’ of a common predicament that was ‘largely ignored by the popular media’. However, Tindall reminded readers that Lethbridge was a ‘highly “emancipated” and indeed unorthodox person’.^49^ Most people did not have access to a decade-long pursuit through private practice, which was a unique experience and a feature in *Baby Blues*. Nonetheless, as historian Tracey Loughran argues, Lethbridge’s experience of feeling both ‘isolated’ and ‘desperate’ was typical of infertile women. This often had a damaging effect on their mental health, especially as most of the ‘blame’ and therefore ‘shame’ focused on women’s and not men’s reproductivity.^50^ As Lethbridge’s experience illustrates, women’s consequent mental health issues could persist despite conceiving a long-awaited child, which often affected their relationships and careers in the long run.

## ‘Drama in crisis’: the BBC, Play for Today and *Baby Blues*

When Lethbridge was disbarred, she became a commissioned scriptwriter during a time of significant change in terms of both class and gender politics within the BBC. This shaped Lethbridge’s approach to writing and later the commissioning of *Baby Blues*. As Lethbridge noticed, which was later confirmed by television historians, the appointment of Sir Hugh Greene as Director General of the BBC and Sidney Newman as head of drama liberated a ‘great swathe of working-class talent’ who would not have had the opportunity under the strict regime of Lord John Reith.^51^ The new ‘talent’ included the Glaswegian director James MacTaggart and producer Kenith Trodd, the pioneers of the original Wednesday PfT. As PfT expert Tom May explains, the series was committed to dramatising socio-political issues in Britain and creative autonomy. As such, MacTaggart and Trodd produced ‘radical’ plays and ‘revolutionised’ television drama’.^52^ As cultural historian Laurel Forster argued, and even Lethbridge herself explained, television producers and directors like MacTaggart were breaking the traditionally rigid style of playwriting and teaching Lethbridge’s generation to write in the style of naturalism and realism, also known as ‘television verité’:

He taught us to forget that you were writing a play and write as we saw life … write as you think … write as you talk … write as you hear … go out into the streets and watch what you see … this was his great contribution.^53^

However, like most prominent institutions in Britain, sexism was rampant for women working at the BBC, though it had made some improvements by the early 1970s and, in turn, determined women writers, actors and producers were slowly overcoming workplace discrimination.^54^ A few producers and directors included more women’s topics and issues. MacTaggart, in particular, was known to ‘defend’, be ‘warm-hearted’ and encourage many up-and-coming women writers, including Lethbridge.^55^ At the time, Lethbridge was one of the 13 per cent of women who wrote PfT scripts and 13 per cent who wrote television drama in general.^56^ Before *Baby Blues*, Lethbridge was not typical of other women writers as her former PfTs mocked both the law system and men’s professional ‘public spheres’ in England, based on her experience as a barrister.^57^ However, *Baby Blues* focused on the female experience of familial strife in domestic spaces and was therefore representative of other women-authored PfTs (like Julia Jones’ *The Piano* (1971), *Still Waters* (1972), *The Stretch* (1973) and *Back of Beyond* (1974)). It is crucial to note that Lethbridge’s aristocratic background and subsequent long-term friendship with MacTaggart perhaps helped her push for a more radical PfT when compared to other women playwriters. Lethbridge’s new focus on women’s domestic experiences, through *Baby Blues*, reflected the WLM’s challenging of family-based gender inequalities through their sentiment of ‘the personal as the political’ to support women.^58^

Nonetheless, when the first and only female producer, Irene Shubik, ran the first era of PfT (1970–1973), she rejected *Baby Blues*. Shubik produced The Wednesday Play and PfT from 1967 to 1972, sharing the role with Graeme McDonald. Shubik was a pioneering television producer who could be ‘difficult’ and ‘cantankerous’ but perhaps had to be when facing strong opposition from male producers in the 1960s and 1970s.^59^ Nonetheless, everyone agreed she had ‘unerring instincts’ regarding drama. Although Shubik enjoyed proving television executives wrong by turning their disinterests into successes, she saw no hope for *Baby Blues*.^60^

In 1969, Lethbridge, when pregnant, signed a contract to submit a play to Shubik on the infamous Kray twins. Shubik was keen to produce this play because Lethbridge had successfully defended the twins in court. Lethbridge, however, experienced a difficult pregnancy, developed postnatal depression and psychosis, divorced O’Connor, and therefore missed the extended deadlines to submit the script.^61^ In 1971, Shubik sent a letter to Lethbridge accepting that the commissioned play could not be finished.^62^ In handwritten apologies to Shubik, Lethbridge explained how she was suffering a ‘fairly lousy time’, including ‘three times back to hospital (redaction)’, which was impairing her creativity and progress.^63^ The promised Kray Twins script was not produced, and in August 1971, *Baby Blues* instead landed on Shubik’s desk, endorsed by MacTaggart.^64^ Lethbridge could not remember how, where or what she used to write *Baby Blues*. All she remembered was that she needed to write it and only recovered from her depression when finishing the play’s first draft. For Lethbridge, writing about a woman who drowns her baby was ‘self-therapy’, a form of ‘catharsis’. It helped her ‘w[a]ke up’ to find her ‘super son’, suddenly two years old.^65^

Shubik, however, was not interested in *Baby Blues*. She could not take ‘seriously’ the ‘uneasy combination of strong [melo-]drama … [and] light-weight sub-plot which almost belong[ed] in light entertainment’. The mother’s mounting depression and final despair, in contrast with her ‘hearty’ husband, supposedly created an ‘uneasy combination’.^66^ Lethbridge responded that she understood *Baby Blues* ‘need[ed] a lot of rewriting’, defending that MacTaggart had at least explained how to improve the script. Lethbridge then maintained that the contrasting characters and styles were based on real life and demanded the script back.^67^ Shubik sent a letter to Ben Travers, the BBC’s Assistant Head of Copyright, rejecting *Baby Blues* for baring no resemblance to her commission and because, in her opinion, it was ‘a very poor piece of women’s magazine writing’.^68^

Three months later, in December 1971, as advised by MacTaggart, Lethbridge sent the script to Shubik’s successor, Graeme MacDonald, who also rejected the play.^69^ Simon, an unknown employee of the BBC, also reviewed the script and responded with misogynistic and unsympathetic opinions. He understood the husband’s adultery and the wife’s ten years of infertility but found the husband ‘quite likeable’ and therefore could not understand ‘what on earth [was] wrong with this woman?’ Simon also wanted the script to ironically show how the husband’s money was funding both his ‘mistress’s abortion and his wife’s childbirth’. Unlike Shubik and MacDonald, Simon concluded that Lethbridge should revise the script by cutting down on the minor characters to develop the main characters better.^70^

Aside from the rejections of *Baby Blues*, 1972 was another challenging year for Lethbridge. Travers cancelled more commissioned plays that Lethbridge failed to deliver, demanding back all advance payments to the BBC.^71^ The altercations between Shubik, Travers and Lethbridge illustrate the lack of support for new mothers who had professional careers in the 1970s, even from other women and BBC workers in general.^72^ Moreover, Shubik’s, Travers’ and also MacDonald’s and Simon’s responses to the script illustrate the ongoing tensions and contradictions within the BBC. As Forster highlighted, the PfT writers and directors wanted to raise debates on current socio-political issues, including ‘gender, families, the domestic, and [ironically] the working environment’. Yet, some producers, especially Shubik, were still cautious about PfT appearing too radical, controversial, and offensive because it sometimes led to outraged responses from television viewers.^73^ Moreover, Shubik, less secure in her role, had to avoid more risky productions as a woman producer in comparison to her male competitors.^74^

Likely less constrained than Shubik, MacTaggart commissioned *Baby Blues* in November 1972 when he returned as director, and Trodd as producer, for what would be marked as the second era of PfT (1973–1977).^75^ Lethbridge attributes the BBC’s production of *Baby Blues* to MacTaggart’s support. Lethbridge acknowledged that *Baby Blues* was ‘raw and sensitive’, but MacTaggart helped her turn it into an ‘[effective] play of sorts’.^76^ During its three-day production, the tension was ‘electric’ as the team thought they had achieved something ‘marvellous’.^77^ The 75-minute play aired between 9.25p.m and 10.40p.m on Thursday, 6 December 1973, on BBC1.^78^

## Baby Blues

When communicating with Lethbridge, she explained how she based many personality aspects of the main protagonist, Lady Lavinia Pointing (played by Zena Walker), on herself.^79^ Lavinia constantly depreciates herself with proclamations that she is a ‘rich and overprivileged bitch’ with a ‘crusading [moral] spirit’. Moreover, Lavinia guiltily mutters that her ‘precious baby’ (underscored in the script) and herself are ‘spoiled’ during confinement.^80^ For Lethbridge and many others, a ‘precious baby’ was—and still is—defined as a baby who requires a lengthy wait, carries significant risks or has been induced by medical treatment.^81^ Like the character Lavinia, Lethbridge was deemed to have a ‘high risk’ pregnancy; both were aged thirty-seven, considered ‘elderly’ first mothers and confined to a private hospital for seven months of pregnancy.^82^ Lethbridge also based the responses from Lavinia’s doctors on what her own doctors said. For example, both Lethbridge’s and Lavinia’s doctors suggested that their keenness, yet difficulty conceiving contributed to the development of maternal mental illness.^83^ The play also suggests that Lavinia’s unsupportive husband—the ‘thirteenth baronet’ Sir Dominic Pointing (played by Norman Rodway)—worsens Lavinia’s mental health by loudly attracting everyone’s attention, even though Lavinia is the main protagonist and is suffering.^84^

Although Lethbridge based this aspect of Dominic’s personality on her former husband, O’Connor,^85^ she deliberately made the protagonists aristocratic, knowing that if viewers watched a play about ‘poor people’, they would stereotype working-class people and attribute maternal mental illness to ‘poverty’ or ‘bad housing conditions’, trivialising the impact of depression and abusive relationships.^86^ She also asserted that *Baby Blues* was not a ‘granddaughter’ of *Cathy Come Home* (1966); monetary donations would not fix maternal mental illness because the illness was a medical problem.^87^ Lethbridge wanted to present a clear message that postnatal depression and psychosis could happen to anyone, regardless of class, wealth or access to support.

The roles and representations of medical professionals were also crucial in the scripting of *Baby Blues*. They reflected Lethbridge’s—and many mothers at the time—real-life experiences of negligent doctors during childbirth and maternal mental illness. For example, the most senior and experienced gynaecologist, Mr Leo Crowley (Sidney Tafler), is presented as a jolly yet ‘practical physician’ who does not take his or anyone else’s life seriously. Crowley practised in an emotionally detached manner and encouraged other doctors to do the same.^88^ This was reflected in Lethbridge’s personal experiences. As explained in her *Spare Rib* article, Lethbridge’s first gynaecologist dismissed her when she asked for support.^89^ In oral histories held at the British Library, many women experienced similar situations when they suffered from maternal mental illness and asked their doctors for help in the 1960s and early 1970s.^90^

In contrast, the younger doctor in *Baby Blues*, Dr Paul Ingram (Robin Hunter)*,* cares about Lavinia’s well-being and regularly visits and converses with her. Ingram also flirts with Lavinia, suggesting an ulterior motive and negating his sincerity in care and support.^91^ Ingram, nonetheless, appears to have reflected the ‘sensible and sensitive G.P’ (General Practitioner) that Lethbridge experienced and acknowledged in her *Spare Rib* article; he was the only doctor who ‘gave [her] hours, weeks, and months of his time and conversation, and saved [her]—and [her] baby’s—life’.^92^ At the time, pharmacology treatments, including anti-depressants and sedatives, were typically prescribed to cure maternal mental illness.^93^ Although being listened to did not save Lavinia in *Baby Blues*, it did appear to alleviate her loneliness and suffering. Therefore, Lethbridge was likely presenting another clear message about the value of talking to someone to improve mental health. Moreover, she later made being heard and listened to the main part of her DA support group.^94^

The other key characters in *Baby Blues* were the four nurses who, again, reflected the varied but well-evidenced stereotypes other women had experienced when delivering a baby in a hospital.^95^ In *Baby Blues*, Nurse Ezrah is the only kind nurse (Claudette Critchlow). In contrast, the more impartial night nurse (June Watson) shames Lavinia when she struggles to get her baby to sleep. Finally, the other two nurses are callous throughout. Sister Maidenhead (Rosalind Knight), for example, is an ‘icy’ nurse and is likened to [Anne] ‘Doris Karloff’ [Widdecombe] in *Baby Blues*.^96^ Karloff was an infamously ‘loveless’, ‘cold’ and ‘hard as nails’ politician who rarely showed empathy towards people, especially women.^97^ As she explained when I corresponded with her, Lethbridge based these characters on the cruel nurses at her private clinic; they looked down on Lethbridge because O’Connor was working-class.^98^

Lethbridge’s play also highlighted the medicalisation and rituals surrounding childbirth. The first scene introduces a pregnant Lavinia on a hospital bed in a gown, in her private room, surrounded by the day nurses and doctors. Her unsupportive husband, of course, is not present. In the first scene, Lavinia expresses her apprehension about her planned caesarean section delivery.^99^ For Lethbridge and the auto-fictional Lavinia, this type of birth would contribute to a ‘sense of alienation’ and that something ‘was wrong’.^100^ As Lethbridge explained to the press, she felt that her baby had not been ‘properly’ born.^101^ In *Baby Blues*, the doctor, Crowley, scolds Lavinia for watching too much television and reading Dr Grantly Dick-Read. Dick-Read influenced many women’s notions of an ideal birth experience, and from the 1930s until his death in 1959, promoted natural childbirth and the practices of birthing breathing techniques and exercises to avoid major medical intervention, much to the exasperation of many midwives and doctors.^102^ Dick-Read also instilled confidence in mothers to follow and act on their mental and bodily intuition during their births.^103^ In *Baby Blues*, Crowley patronisingly tells Lavinia to ‘leave it to the professionals and stick to reading [British women’s fashion magazines] *Vogue* and *Harper*’. Lavinia defends herself and states that she’d ‘still like to know what’s happening to my body—and [underlined in the script] my baby’. This scene reflected the typical rebuke women received when trying to regain ownership and control over their bodies in medical spaces during childbirth. Moreover, Lavinia communicates—and potentially teaches—viewers how to challenge their doctors.^104^

The halfway point of *Baby Blues* features the scene that produced the most outrage from viewers: a 1 minute and 24 second film of a real caesarean section. This scene leads to the second half of the play, which focuses on Lavinia’s mental decline and stresses the caesarean's significance as a contributing factor.^105^ As explained in the *Spare Rib* article, Lethbridge again based this on her experience. After the caesarean, Lethbridge felt like the ‘happiest woman in the world’ for the first five days, but then ‘went out of [her] mind’.^106^ In *Baby Blues,* Lethbridge imitates this through Lavinia's emotional transitions from euphoria to hysteria and depression. Lavinia drifts in and out of sleep, refuses food and becomes convinced that something is ‘wrong’ and that she has ‘the wrong baby’. The unkind nurses dismiss her, affirming that ‘everyone feels that way after a C-section’.^107^

Lethbridge regularly cried for the first eighteen months post-birth and constantly thought about suicide.^108^ Her first gynaecologist explained that this was normal, nothing more than a ‘touch of the baby blues’ and dismissed her.^109^ Lethbridge shares this experience through a conversation between the two doctors in *Baby Blues*. Crowley reassures the character Lavinia that ‘puerperal depression’ is common after a caesarean and only lasts one or two weeks. He also tells Lavinia that she can leave the hospital if she has support at home. Ingram, however, insists that Lavinia needs treatment (for instance, anti-depressants). The older and more authoritative Doctor Crowley contests and the young Doctor Ingram abides.^110^ This might be intended to suggest that the next generation of doctors would be more sympathetic to women’s needs or that the aspiration to offer patients additional care and compassion declines throughout a doctor’s career. It also illustrates how a woman’s health experience could drastically vary depending on their allocated doctor.

Moreover, *Baby Blues* featured another factor commonly claimed by doctors and women sufferers as contributing to maternal mental illness; insensitive comments made by medical professionals, family, and friends, which induced feelings of guilt and uselessness in new mothers.^111^ When Cowley first visits Lavinia after the birth, he comments on her ‘lousy pregnancy’. When Lavinia’s son develops an infectious illness and needs to be separated from the other babies, Lavinia is blamed. When Lavinia fails to soothe her crying baby in an isolated room, the night nurse criticises Lavinia again. Finally, when Lavinia arrives home, the ‘daily help’ upsets Lavinia by boasting about her own ‘nine-pound baby and dry labour’.^112^ As Lethbridge explained when corresponding with her, she satirised her own experiences of when people made her feel inadequate about the pregnancy, the childbirth experience, and her ability as a new mother.^113^ This was—and still is—cited as a critical factor and cause of maternal mental illness within women’s testimonies.^114^

Like other mothers who suffered psychosis, both Lethbridge and Lavinia experienced voices in their heads and hallucinations.^115^ It is important to note that this condition was rarer than postnatal depression alone and often posed a greater risk of infanticide when left untreated, as *Baby Blues* illustrated.^116^ In *Baby Blues*, each time Lavinia hears, ‘When we are born, we cry that we are come to this great stage of fools’ from the theatre production *King Lear*, Lavinia’s mental health deteriorates further. Finally, one of the last scenes shows Lavinia almost drowning her baby. While the voices in her head repeat ‘Fear death by water’ from ‘The Burial of the Dead’ part of T.S. Eliot’s *The Waste Land* poem, a crazed Lavinia holds her baby’s head just above the bath water. The scene cuts when Lavinia shouts for help and leaves. However, Lavinia eventually picks up her baby from the nursery and carries him to the bathtub. The final scene shows an empty jar of medication and a lifeless Lavinia and baby on the bed, suggesting rather than confirming infanticide and suicide.^117^ This ambiguity was deliberate on such a taboo topic.^118^ Nonetheless, the desire by mothers to harm their new-born babies when mentally unwell, even if they fought against it, was more common than the public was ready to acknowledge.

## The first (negative) and second (positive) wave

*Baby Blues* enjoyed high viewership numbers on its transmission, with roughly 13.8 per cent of Britain's population tuning into BBC1 for the programme. This equated to approximately 7.3 million people, exceeding the average viewing figures for PfT.^119^ BBC1 was also the most widely watched television channel, making *Baby Blues* a provocative discussion point in newspapers alone for months if not years.^120^

During the first week-long wave of responses, the critics, writing in both right and left-wing newspapers, ridiculed the play. All the critics acknowledged that Lethbridge had suffered the same condition as the protagonist and that it helped her recover.^121^ However, almost all male critics belittled maternal mental illness as a condition. Even before it aired, male critics mocked *Baby Blues* as ‘a story of a woman who, after struggling to get a baby, then wants to get rid of him’.^122^

Most of the critics also despised *Baby Blues’* style. Russell Davis and John Bull (*New Statesman*) described *Baby Blues* as ‘a shrieking tract on … puerperal depression’ and ‘melodrama … at full blast’.^123^ Sean Day-Lewis (*Daily Telegraph*) assumed that Lethbridge did not want to be a ‘bore’ on the subject, having suffered from it herself, and therefore submerged the topic in ‘surrounding caricature’.^124^ In her previous plays, Lethbridge had used tragicomedy caricature to mock the law system in England, much to viewers’ delight.^125^ As such, Lethbridge perhaps thought that it was the best way to draw attention to the issues surrounding childbirth and the suffering of mothers, mock the dismissive medical professions, and get such a taboo topic on television in the early 1970s.

Most critics also lacked sympathy towards the emotional turbulence—and physical changes—that mothers might endure during and after childbirth. Michael Ratcliffe, writing in *The Times*, for example, initially found Lavinia ‘amiable’ but became frustrated when she became ‘obsessed by the manner of [the baby’s] birth, [and] by her own uselessness*’*. He also did not understand why Lavinia remained married to Sir Dominic.^126^ Similarly, James Thomas’s piece in the *Daily Express* did not believe that an ‘intelligent woman’ would ‘suffer … all the idiot indiscretions of her husband … in such a time of crisis’.^127^ As the critics could not accept that an amiable and intelligent woman could be with an aristocratic drunk, the play was not realistic to them. As Alana Harris’s and Timothy Jones’ edited collection on *Love and Romance in Britain* illustrated, this contrasts with the lived experience of women across all social backgrounds who have stayed in unhappy marriages for decades despite emotional, psychological, and physical abuse or because of religious beliefs, the stigma of divorce, lack of alternative options, safety reasons, or simply because they were in love and believed their partners would change.^128^

The critics, like Ratcliffe, were also unsympathetic to Lethbridge’s focus on private healthcare, which highlighted some men’s ignorance of the desperate costs and measures that women would go to conceive when being child-free was taboo.^129^ Davis and Bull added that *Baby Blues* had now endowed a ‘brand-new nervous disposition’ for many thousands of ‘previously unflappable viewers’, which, in turn, trivialised maternal mental illness.^130^ Finally, the ambiguous ending annoyed Ratcliffe.^131^ He lacked sensitivity towards the century-long ‘pain, shame and fear’ sentiments surrounding infanticide (and suicide) and was oblivious to Lethbridge’s constraints as a female playwright.^132^

However, the most significant controversy surrounding *Baby Blues* was the caesarean birth; complaints from viewers jammed the BBC switchboard across Britain.^133^ The critics acknowledged that the caesarean delivery was a significant contributor to Lethbridge’s and Lavinia’s puerperal depression, as it might be for many other women—and was a crucial aspect of *Baby Blues*. Nonetheless, most critics, like Thomas, did not think viewers needed to see the procedure.^134^ Viewers had been similarly ‘squeamish’ when a caesarean section was shown a decade before, in 1963, on the BBC’s *Your Life in Their Hands*.^135^ The ‘Clean-up TV’ campaigner Mary Whitehouse used the scene from *Baby Blues* to attack the BBC and her ‘real life nemesis’, the BBC Director-General Hugh Green.^136^ The outraged Whitehouse confronted the BBC Chairman Michael Swann for not including *Baby Blues* in the BBC’s annual report on what could be considered obscene on television or radio.^137^ Whitehouse, the general secretary of the National Viewers’ and Listeners’ Association, was a fierce defender of conservative family values and ‘moral pollution’.^138^ Through national newspapers across Britain, Whitehouse slandered the play as ‘Obscene!’, ‘Blasphemous!’, ‘Insensitive!’ and ‘Irresponsible in handling post-natal depression and caesarean birth’. She argued that the implied infanticide and suicide in *Baby Blues* might influence women going through similar experiences to do the same. Although many people did not take Whitehouse seriously,^139^ she undermined Lethbridge’s attempt to raise awareness, understanding and support for maternal mental illness by asserting, ‘WOMEN DON’T DROWN THEIR BABIES’. Whitehouse also thought the depiction of a caesarean birth would disturb mothers who were undergoing the same operation.^140^ Some viewers, however, challenged Whitehouse. Mrs Steed of Buckinghamshire, for example, sent a letter to the *Daily Mail*, suggesting that it was about time Whitehouse ‘took stock of her own mind and outlooks’ because the birth in *Baby Blues* was authentic and not ‘insensitively explicit’.^141^

As *Baby Blues* had elicited public controversy, there were apologies and inquests at the BBC executive level.^142^ Nonetheless, the Television Service merely advised the BBC not to show scenes like a caesarean section for the time being. Moreover, the opinion of the Television Service during a Board of Governors meeting was ‘deeply divided’; half thought the scene was justified and the other half saw it as unnecessary. However, the Director of Programmes, Mr Milne, was confident that the scene in *Baby Blues* was not ‘obscene or indecent’.^143^

Perhaps uncoincidentally, two of the only three critics empathetic to Lethbridge and *Baby Blues* were women. Liz Cowley (*Evening Standard*) had personally experienced two caesareans and ‘the blues’ herself and strongly applauded Lethbridge’s play.^144^ Elizabeth Allen (*The Stage* and *Television Today*) had understood and supported Lethbridge’s message through *Baby Blues* that puerperal depression can affect anyone and that support networks are vital for the mother’s and baby’s survival. She defended the ‘caricature’ characters, arguing that this is how they would have appeared to the suffering mother. Allen also affirmed that unkind nurses ‘*do* exist’ in maternity wards (underscored in the article) and praised the production team for not downplaying the intense and difficult theme.^145^ Finally, Shaun Usher (*Daily Mail*) was the only self-aware male critic who sympathised with how ‘rotten’ some men could be and how women have a tough time. He was grateful for the opportunity to try and identify with a pregnant woman and new mother through the character of Lavinia and did not belittle how maternal mental illness could feel for a woman.^146^

Albeit based on a small sample of 106 people, the BBC’s audience research report further illustrated *Baby Blue*s’ extremely mixed reception. One-third of people ‘had nothing but praise’; one-third had ‘various reservations’, and the remainder were either bored or strongly disapproved of the play. The positive and enthusiastic viewers described *Baby Blues* as ‘extremely interesting’, ‘unusual’, ‘moving’ and ‘absorbing’. Some were particularly impressed because it was ‘real’ and ‘true to life’ in its characters, situations, and setting. They described some sequences as ‘marvellously lifelike’, particularly the delivery by caesarean section.

In contrast, the viewers who moderately rated *Baby Blues* objected to the ‘too exaggerated situations’ and ‘caricatured presentations of the hospital staff’. The viewers who disliked *Baby Blues* remarked that it was ‘rather boring and slow’ or that maternal mental illness had been presented as ‘distasteful’. Some also thought the picture of the hospital was ‘completely distorted and unauthentic’ and that the tragic ending was unnecessary. A small group were indignant, describing the play as ‘most depressing’ and ‘one of the foulest’ they had ever seen or either ‘disgusting; an insult to the medical and nursing service’ and ‘squalid and offensive; a disgrace to all concerned’.

The play considerably impacted some viewers; one, for instance, felt ‘sad and shaken’, whereas another found it ‘too disturbing to be enjoyed’ but still highly rated it. For many, but not everyone, the hospital atmosphere was also convincing in every detail.^147^ Clearly, like the critics, most of the first wave of responding reviewers were not comfortable with a taboo topic like maternal mental illness or the graphic depiction of birth, the raising of questions around medical care and familial support, and the potentially fatal consequence of postnatal depression or psychosis.

Nonetheless, as *Baby Blues* had ignited major publicity on the issue of postnatal depression, a second, more positive and longer-lasting wave of supportive letters arrived in Lethbridge’s letter box. Two months after the play aired, Lethbridge reported that she had received supportive letters from doctors, nurses, and 203 depressed mothers either thanking her or pleading for ‘help’.^148^ Within the first year of *Baby Blues*’ transmission, Lethbridge received three thousand letters from people suffering from postnatal depression.^149^ Lethbridge believed that each letter, from all parts of Britain, offered ‘mini-catharsis for the suffering writer’, similar to how she felt when she wrote the script for *Baby Blues*. Some of the admissions in the letters were extreme, yet perhaps contributed to the de-stigmatisation of maternal mental illness for women by normalising discussions on the matter. The responses attest to women feeling comfort and less guilt in knowing they were not suffering alone and that their experiences were being publicly listened to for the first time. One woman in Surrey wrote, ‘I have a kind husband, a beautiful baby, no financial problems and still I want to die’. In Aberdeen, a woman felt reassured: ‘I thought I must be the most wicked person in the world to have thoughts like these’. Another in Cumberland confessed, ‘I put a pillow on my baby’s face and climbed a mountain 4,000 feet high’. And finally, another woman in London professed, ‘I battered my baby’s head against the wall, repeatedly, and it wasn’t until we got him to hospital that we realised he was dead’. For Lethbridge, these letters justified the ‘whole dreadful business’ of harsh critics and everyday complaints. She also respected people’s confidentiality unless she was ‘specifically released from doing so’.^150^

Moreover, ordinary people, inspired by the play, started to send letters to newspapers with suggestions on how hospitals and voluntary organisations could offer better support to prevent maternal mental illness. Mrs Stoke writing to the Sunday Telegraph, for example, advised hospitals not to discharge mothers as soon as their babies were born and that a voluntary scheme of older and more experienced women, acting as ‘temporary nannies’, could support new mothers for a few hours every day.^151^ Some medical experts also wrote to Lethbridge in support of her play. One psychiatrist, for instance requested a copy of the videotape to show medical students at a London teaching hospital to explain the condition to students after complaining about how difficult this was to convey.^152^ Lethbridge, however, wanted to do more to support sufferers.

## Depressives Anonymous

Lethbridge founded Depressives Anonymous (DA) in the Spring of 1973 to offer long-lasting support for mothers who had self-diagnosed or been medically diagnosed with any form of maternal mental illness.^153^ She started with a hotline for people to speak to non-medical professionals who had suffered themselves. When Lethbridge had been unwell, she telephoned the Samaritans for support and was greeted by such a ‘hearty rugby-playing young muscular Christian’ that she was speechless. Consequently, like Alcoholics Anonymous, she wanted to limit membership to present and former sufferers. The first three local groups were in Dorset, East Anglia and Yorkshire, and by asking for publicity through *Spare Rib*, Lethbridge (and other women) set up another twenty-six groups across Britain by late March 1974.^154^ By this time, Lethbridge expanded DA to include in-person meetings for both women and men who were ‘depressive’ in general; the groups were not designed to necessarily ‘cure’ depression but to ‘lessen guilt and isolation and to help the sufferer live with the problem’.^155^

In Autumn 1974, via *Spare Rib*, Lethbridge announced DA’s first national conference to speak about women’s and men’s depression*.* The Church of St Mary-le-Bow in London hosted the event on Saturday, 28 September 1974. Lethbridge invited a ‘distinguished' female psychiatrist to speak at the conference and the meeting started with a viewing of *Baby Blues*. The event received significant support from men with over 150 attending.^156^ Jean Robinson then explained the inadequacies of NHS provision in relation to maternity care. From 1973 to 1975, Robinson was the Chair of the Patients Association and later became President of the Association for Improvements in the Maternity Services (AIMS).^157^ Keith Middleton, who organised the small group meetings in Birmingham in 1974, then spoke about the need for a Charter of Rights for Mental Patients because of the discrimination in employment and life insurance against depressives who had been hospitalised. Finally, the conference aimed to set up more groups nationwide, which proved successful.^158^

By November 1974, a month after the conference, DA expanded and had groups in Bristol, Swindon, Trowbridge, Weymouth, Dorchester, Portland, Andover, Southampton, Hastings, Hook, Portsmouth, Havant, Winchester, Crawley, Horley, Redhill and Dorset. As Lethbridge struggled to support her two young children as a single mother in the wake of divorce and consequent bankruptcy, other women stepped into leadership positions within DA. Janet Stevenson, for example, with her ‘immense energy … determination’ became the leader and key figure of the DA and prepared the newsletters.^159^ The psychologist Dorothy Rowe was another key figure who medically endorsed DA and similar self-help groups in the future. When attending the sessions with her depressed clients, she realised that most members benefitted from being heard and listened to, not from being prescribed anti-depressants and Electroconvulsive therapy (ECT) by psychiatrists. The group influenced her ideas and her writing *Depression: the Way Out of Your Prison* (1983), which won the Mind book of the year award in 1984. Rowe attributes DA to the development of other self-help organisations, such as the *Users of the Psychiatric System* and the *Hearing Voices Movement*. She affirmed that ‘the biggest change in our understanding of severe mental distress [came] from the users of the psychiatric system, not the professionals’.^160^ Another DA linchpin was Ann Gaines, who led the meetings for three groups in London (East End, North and South) and stressed the need for more day-time helpers to support housemakers like herself.^161^ Through *Spare Rib*, Gaines praised DA but wanted to address London’s ‘peculiar problems’ because of its size. She was concerned about the risk of jealousy from other voluntary groups, like The Samaritans, who might feel DA was ‘poaching on its territory’. She innovatively recommended that DA research who and where these groups were, what they did, and how they could help, suggesting personal visits and discussions to start ‘on the right footing’.^162^

In Spring 1975, according to an article in the *Guardian*, the two major features of DA were an ‘informal atmosphere’ and a ‘tight-knit group’ of people who could talk freely. DA likened this to an ‘extended family, in which all members form strong bonds’. DA, organised by ‘depressives’, also provided ‘mutual help’ through a ‘loose framework’ of fortnightly meetings rather than rigid therapy. A ‘helper’ who had recovered from or had their illness under control was believed to be better equipped to support other sufferers. DA defined a ‘depressive’ as someone who felt isolated, inadequate, disorientated, suicidal or struggled to make decisions, had memory lapses, and lacked concentration. The organisers explained how depression could affect anyone and did not discriminate based on gender, age, class and intelligence.

The article also asserted that there was ‘no clearcut reason’ for depression and that it was essential to understand how separate categories of depression were self-defeating and detracted from the self-respect that depressed people needed. In the future, DA wanted to work alongside the medical profession, social services, and other voluntary bodies. By 1975, they worked with The Samaritans, who referred their callers and clients to DA. DA was also trying to acquire charity status—which they achieved in 1979—and welcomed donations.^163^ The 1975 article concluded with the sharing of three distinct experiences of depression from different people, which continued Lethbridge’s trend of using personal narratives to reduce stigma and grow people’s confidence in asking for help.^164^

By the summer of 1975, DA had started to conduct surveys to research the causes of postnatal mental illness. For example, the results from a study of one thousand postnatal depression sufferers revealed a strong link to artificial birth methods (induction).^165^ By 1977, a woman in Edinburgh even set up a ‘postal depressives anonymous’ to support women who mentally struggled to attend or could not travel to DA meetings.^166^

In 1980, one of the first personal accounts of postnatal depression, *Postnatal Depression* (1980), by playwright and journalist Vivienne Welburn, even dedicated her acknowledgements to DA, and advised other sufferers to join DA for support as it had helped her in the mid-70s.^167^ In 1980, Depressives Anonymous changed their name to Depressives Associated. This was in-trend with the watershed of literature on postnatal depression by 1980, where testimonies, like Welburn’s, were less likely to be anonymised in an attempt to take ownership and further de-stigmatise depression.

## Conclusion

*Baby Blues* became a part of the broader changes in maternal mental illness discourse and understanding, and Lethbridge was one of the many vital figures assisting this change. This article has traced how, even before the commissioning of *Baby Blues*, Lethbridge ‘fierce[ly]’ explored challenging subjects within women’s lives in the public domain, aiming to raise awareness and support.^168^ As illustrated in this article, for example, she published articles on the struggles of infertility in both the mainstream media and feminist literature.

This article also demonstrated how Lethbridge’s feminist and left-wing sentiments were a part of the wider WLM movement concerned with the medicalisation of childbirth and reproductive rights. By extension, this influenced the growing tensions in class and gender dynamics within the BBC and, more notably, the PfT series through its experimentation with a ‘radical’ play on motherhood and mental health. By tracing the play’s reception, this article illustrated how television shaped public discourse and cultural changes on maternal mental illness. Lethbridge’s experience as a scriptwriter for the BBC while suffering from life-threatening mental health issues also illustrated the lack of support from media production companies.

Although many reviews of the play revealed that *Baby Blues* was not of its time, hence the controversy it provoked, it prompted a campaign to develop a network of self-help groups for depressed people. DA is a historically neglected but important grass-roots organisation that focused on the patients’ rather than the medical professional’s voice from the mid-1970s onwards.^169^ DA was also part of a more significant shift in the rise of self-help organisations in England, which again reflected the ethos of the WLM in its challenging of medical professionals who dismissed women’s mental health issues.^170^ Women like Lethbridge, Stevenson, Rowe, Gaines and Welburn, along with many others whose names were not recorded in the archive, were vital to DA’s success and the support it offered for mothers in need. Today, half a century after it was set up, this support group—with its focus on sufferers speaking to non-medical people who have recovered or at least cope with their own mental illness—still exists, now known as Depression UK.^171^

Lethbridge alone, however, was not responsible for this movement in drawing attention to maternal mental illness. To begin with, MacTaggart encouraged the BBC’s spotlight on the mental health politics of motherhood in Britain for the first time on television, despite other directors rejecting *Baby Blues*. Other women who Lethbridge worked with and was inspired by, like Sheila Kitzinger, a childbirth educator, and Katharina Dalton, a doctor who pioneered postpartum hormone replacement therapy, fought for mothers’ reproductive and childbirth agency in different ways to Lethbridge.^172^ Moreover, many others supported and promoted the long-lasting DA. These included the thousands of people who sent letters and Robinson, Stevenson, Rowe, Gaines and Welburn. Welburn, in particular, was one of many who built on Lethbridge’s auto-fictional narrative by publishing her own experiences of postnatal depression in 1980.^173^ Clearly, Lethbridge, through *Baby Blues,* helped pave an opening for maternal mental illness narratives that could no longer be closed.

## Notes

1 Nemone Lethbridge, ‘Not So Much Childbirth as Depression’, *Radio Times*, January 4, 1974, 76; *Baby Blues*, ‘Play for Today’, BBC1, December 6, 1973.

2 Dorothy Rowe, ‘Play for Today Shows Art Can Change Life’, *Guardian*, April 9, 2008.

3 Vicky Ball, ‘Women Writers and Writing Women into Histories of Play for Today’, *Journal of British Cinema and Television* 19 (2022): 150–72.

4 Brice Pitt, ‘“Atypical” Depression Following Childbirth’, *British Journal of Psychiatry* 114 (1968): 1325–35; Hilary Marland, ‘‘Drowned in a Sea of Inhumanity’: Natural Childbirth, Postnatal Depression and the National Childbirth Trust, 1956–1980s’, *Social History of Medicine* (2023): 1–24, 1; The NHS currently reports that postnatal depression affects more than one in every ten women within the first year of giving birth, National Health Service, ‘Postnatal Depression’, 4 August 2022. https://www.nhs.uk/mental-health/conditions/post-natal-depression/overview/ (accessed February 22, 2024); The NHS reports that postpartum psychosis affects one in every thousand women within several weeks of giving birth, National Health Service, ‘Postpartum Psychosis’, 18 October 2023. https://www.nhs.uk/mental-health/conditions/post-partum-psychosis/ (accessed February 22, 2024).

5 Nemone Lethbridge, ‘Postscript to Baby Blues’, *Spare Rib*, Issue 21, March 1974, 17.

6 George Stevenson, *The Women's Liberation Movement and the Politics of Class in Britain* (New York: Bloomsbury Publishing, 2019), 3.

7 Sheila Rowbotham, ‘To Be Or Not To Be. The Dilemmas of Mothering’, *Feminist Review* 31 (1989): 82–3; Ball, ‘Women Writers and Writing Women Into Histories of Play for Today’, 163.

8 I am grateful to Lethbridge for giving me the opportunity to discuss her work, during which she confirmed my archival findings.

9 *Newcastle Evening Chronicle, Aberdeen Evening Express, Belfast Telegraph, Daily Mail, Daily Telegraph, Sunday Telegraph, Daily Express, The Times, Sunday Times, Observer, Guardian* and *Sunday People*.

10 BBC WAC, Audience Research Reports, VR/73/695, Play for Today, *Baby Blues*, 4 January 1974.

11 Jane M. Ussher, *The Madness of Women: Myth and Experience* (London: Routledge, 2011), 1–14; Jane M. Ussher, ‘Depression in the Postnatal Period: A Normal Response to Motherhood’, in *Pregnancy, Birth and Maternity Care: Feminist Perspectives*, ed. M. Stewart (London: Books for Midwives, 2004), 105–20, 105.

12 Ussher, *The Madness of Women*, 4.

13 Ibid., 13.

14 Jane M. Ussher, *Managing the Monstrous Feminine: Regulating the Reproductive Body* (London: Psychology Press, 2006).

15 Angela Davis, ‘A Revolution in Maternity Care? Women and the Maternity Services in Oxfordshire c.1948–1974’, *Social History of Medicine* (2001): 389–406.

16 Marland, ‘‘Drowned in a Sea of Inhumanity’’; Angela Davis, *Modern Motherhood: Women and Family in England, 1945–2000* (Manchester: Manchester University Press, 2012), 84.

17 Sue Bruley, ‘‘It Didn’t Just Come out of Nowhere Did It?’: The Origins of the Women’s Liberation Movement in 1960s Britain’, *Oral History* 45 (2017): 67–78.

18 Lesley Doyal, ‘Women, Health and the Sexual Division of Labor: A Case Study of the Women's Health Movement in Britain’, *International Journal of Health Services* 13 (1983): 373–87; Marland, ‘‘Drowned in a Sea of Inhumanity’’, 9.

19 Pitt, ‘“Atypical” Depression following Childbirth’, 1325–35; Ann Oakley, ‘The Sociology of Childbirth: An Autobiographical Journey Through Four Decades of Research’, *Sociology of Health and Illness* 38 (2016): 689–705.

20 Ball, ‘Women Writers and Writing Women into Histories of Play for Today’, 170.

21 D.A.N. Jones, ‘Mother Love That kills’, *Radio Times*, November 29, 1973, 26–7; Lethbridge, ‘Not So Much Childbirth as Depression’.

22 Ball, ‘Women Writers and Writing Women into Histories of Play for Today’, 163.

23 A Women’s Liberation Supporter, ‘Letters: Painless Childbirth’, *Spare Rib*, Issue 20, February 1974, 3; Lethbridge, ‘Postscript to Baby Blues’, 17; Zoe Strimpel, ‘*Spare Rib*, The British Women’s Health Movement and the Empowerment of Misery’, *Social History of Medicine* 35 (2022): 217–36.

24 Lucy Delap and Zoe Strimpel, ‘*Spare Rib* and the Print Culture of Women’s Liberation’, in *Women’s Periodicals and Print Culture in Britain, 1940s–2000s*, ed. Laurel Forster and Joanne Hollows (Edinburgh: University of Edinburgh Press, 2020), 46–66, 55.

25 Lucy Delap, ‘Feminist Business Praxis and *Spare Rib* Magazine’, *Women: A Cultural Review* 32 (2021): 250; Delap and Strimpel, ‘*Spare Rib* and the Print Culture of Women’s Liberation’, 55, 46.

26 Nemone Lethbridge, ‘Post Natal Depression’, *Spare Rib*, Issue 21, March 1974, 17, 1.

27 Frances Horsburgh, ‘A Newspaper Office where Men are Strictly Taboo’, *Spare Rib*, Issue 180, July 1987, 39.

28 Sue O’Sullivan, ‘What Can We Do about the Baby Blues?’, *Spare Rib*, Issue 85, August 1979, 10; Gail Ogbuehi, ‘Racism in Tower Hamlets’, *Spare Rib*, Issue 225, July 1991, 4.

29 BBC WAC, *Woman’s Hour*, ‘Baby Blues’, Radio 4, July 11, 1974.

30 Sarah Crook, ‘The Uses of Maternal Distress in British Society, c.1948–1979’ (PhD diss., Queen Mary University of London, 2016), 60–100; Sarah Crook, *Postnatal Depression in Post-War Britain: Women, Motherhood and Social Change* (Manchester: Manchester University Press, forthcoming).

31 Lethbridge, ‘Postscript to Baby Blues’; Ann Oakley, *Taking it Like a Woman* (London: Jonathon Cape, 1984), 47.

32 ‘Nemone Lethbridge’, *The Light of Experience*, BBC2, January 23, 1982.

33 Jimmy O’Connor, *The Eleventh Commandment* (UK: Seagull S.A, 1976), 174.

34 Catherine Baksi, ‘Legal Hackette Lunches with Nemone Lethbridge’, *Legal Hackette’s Brief*, March 6, 2018.

35 Catherine Baksi, ‘Nemone Lethbridge: ‘It's Impossible for Anyone to Go to the Bar Who Hasn't Got a Rich Daddy’’, *The Times*, March 19, 2020.

36 Nemone Lethbridge, ‘Man on the Moon’, in *Woman on Woman*, ed. Margaret Laing (Sidgwick & Jackson, 1971), 81; Hilary Heilbron, *Rose Heilbron: The Story of England’s First Woman Queen's Counsel and Judge* (Oxford: Hart Publishing, 2012); Nemone Lethbridge, *Nemone: A Young Woman Barrister’s Battle against Prejudice, Class and Misogyny* (Nemone Lethbridge, 2021), 350.

37 O’Connor, *The Eleventh Commandment*, 12, 17, 73, 147, 153, 163.

38 Ibid., 171–3, 91.

39 Lethbridge, *Nemone*, 332, 349–50; Nicholas Tomalin, ‘No Room at the Bar’, *Sunday Times*, March 1, 1964, 31.

40 Ian Jack, ‘The Odd Couple – And What Broke the Spell’, *Sunday Times*, May 2, 1976, 42.

41 Lethbridge, *Nemone*, 356.

42 Tracey Loughran, ‘Infertility Through the Ages – And How IVF Changed the Way We Think about It’, in *The State of Families: Law, Policy, and the Meanings of Relationships*, ed. Jennifer Reich (New York: Routledge, 2020), 230–4, 232.

43 Gayle Davis and Tracey Loughran, eds., *The Palgrave Handbook of Infertility in History* (London: Palgrave Macmillan, 2017).

44 O’Connor, *The Eleventh Commandment*, 173.

45 Lethbridge, ‘Man on the Moon’, 82–5.

46 Nemone Lethbridge, ‘Infertility Rites’, *Observer*, October 24, 1971, 33; Lethbridge, ‘Man on the Moon’, 84–8.

47 Laing, *Woman on Woman*, 3.

48 Lethbridge, ‘Man on the Moon’, 81.

49 Gillian Tindall, ‘Who Enjoys Being a Girl?’, *Guardian*, October 28, 1971, 11.

50 Loughran, ‘Infertility Through the Ages – And How IVF Changed the Way We Think about It’, 233.

51 Lethbridge, *Nemone*, 338; Peter Astley Grosvenor Waymark, ‘Television and the Cultural Revolution: The BBC Under Hugh Carleton Greene’ (PhD diss., Open University, 2006).

52 Tom May, ‘Play for Today: A Statistical History’, *Journal of British Cinema and Television* 19 (2022): 133; Lethbridge, *Nemone*, 338.

53 Laurel Forster, ‘1970s Television: A Self-Conscious Decade’, in *British Film Culture in the 1970s: The Boundaries of Pleasure*, ed. Sue Harper and Justin Smith (Edinburgh: Edinburgh University Press, 2011), 85–98, 92–3; ‘A Tribute to James MacTaggart’, *In Vision*, BBC1, June 7, 1974.

54 Ball, ‘Women Writers and Writing Women Into Histories of Play for Today’, 160; Tom May, ‘Poorly Paid, But Proud to Work in Teams Producing ‘Quality’: An Oral History of Women’s Experiences Working in BBC Drama’, *Critical Studies in Television* 17 (2000): 299–303.

55 Madeleine Macmurraugh-Kavanagh, ‘Boys on Top: Gender and Authorship in the BBC Wednesday Play, 1964–70’, *Media, Culture and Society* 21 (1999): 201–2; Lethbridge, *Nemone*, 353.

56 Ball, ‘Women Writers and Writing Women into Histories of Play for Today’, 151, 154.

57 Before *Baby Blues*, Lethbridge’s plays were *The Portsmouth Defence*, *The Little Master Mind* and *An Officer of the Court*.

58 Ball, ‘Women Writers and Writing Women into Histories of Play for Today’, 162–3; Madeleine Macmurraugh-Kavanagh, ‘Too Secret for Words: Coded Dissent in Female-Authored Wednesday Plays’, in *British Television Drama: Past, Present and Future*, 2nd ed., ed. Jonathon Bignell and Stephen Lacey (London: Palgrave Macmillan, 2014), 191–202, 199.

59 Anon., ‘Irene Shubik’, *Times*, October 9, 2019, 54; Macmurraugh-Kavanagh, ‘Too Secret for Words’; Macmurraugh-Kavanagh, ‘Boys on Top’, 409–25; Irene Shubik, *Play for Today: The Evolution of Television Drama* (Manchester: Manchester University Press, 2001).

60 Anon., ‘Irene Shubik’.

61 Lethbridge, *Nemone*, 335, 337; O’Connor, *The Eleventh Commandment*, 181–2; BBC WAC, Nemone Lethbridge Drama Writer’s File, Commissioning Brief, 10 March 1969 and Irene Shubik Letters (1969/1970).

62 Ibid., Shubik Letter to Lethbridge, 19 March 1971.

63 Ibid., Lethbridge and Shubik Letters, June and July 1971.

64 Ibid., Letter from Pamela Gillis (on behalf of Lethbridge) to Shubik, 4 August 1971.

65 Lethbridge, ‘Postscript to Baby Blues’, 17

66 BBC WAC, Nemone Lethbridge Drama Writer’s File, Shubik’s Letter to Lethbridge, 2 September 1971.

67 Ibid., Lethbridge’s Letter to Shubik, 7 September 1971.

68 Ibid., Shubik’s Letter to Ben Travers, 9 September 1971.

69 Ibid., Lethbridge’s Letter to Graeme McDonald, 12 December 1971, and McDonald’s Letter to Lethbridge, 18 January 1972.

70 Ibid., Simon’s Letter (1971/2).

71 Ibid., Ben Travers Letter to Pamela Gillis (on behalf of Lethbridge), 10 February 1972.

72 May, ‘Poorly Paid, But Proud to Work in Teams Producing ‘Quality’, 299.

73 Forster, ‘1970s Television’, 97.

74 Irene Shubik, ‘Television Drama Series: A Producer’s View’, in Bignell and Lacey, *British Television Drama*, 45–51; Ruth McElroy, ‘Women in Television Drama: Voices from the Workplace’ in *Television for Women. New Direction*, ed. Rachel Moseley, Helen Wheatley, and Helen Wood (London, New York: Routledge, 2017), 35–43.

75 BBC WAC, Nemone Lethbridge Drama Writer’s File, Ben Travers’ Letter to Nemone Lethbridge, 3 November 1972.

76 ‘A Tribute to James MacTaggart’, *In Vision*.

77 Lethbridge, ‘Postscript to Baby Blues’, 17.

78 Anon., ‘The Television Guide’, *Daily Mail*, December 6, 1973, 30.

79 Correspondence with Nemone Lethbridge, 19 June 2023.

80 *Baby Blues*.

81 Howard Minkoff and Richard Berkowitz, ‘The Myth of the Precious Baby’, *Obstetrics and Gynecology* 106 (2005): 607–9.

82 *Baby Blues*; Lethbridge, ‘Man on the Moon’, 88.

83 *Baby Blues*; Correspondence with Nemone Lethbridge, 19 June 2023.

84 *Baby Blues*.

85 Lethbridge, *Nemone,* 333; O’Connor, *The Eleventh Commandment*, 190.

86 Lethbridge, ‘Not So Much Childbirth as Depression’, 76.

87 Jones, ‘Mother Love That Kills’, 26–7.

88 *Baby Blues*.

89 Lethbridge, ‘Postscript to *Baby Blues*’, 17.

90 British Library Oral Collection, C900/06552, 1998.

91 *Baby Blues*.

92 Lethbridge, ‘Postscript to *Baby Blues*’, 17.

93 British Library Oral Collection, C15593101 050, 2012; C905A 28 01, 1999. C905A 42 01, 1999; C905A 39 01, 2000.

94 Depressives Anonymous, ‘Self-Help’, *Sunday Times*, March 24, 1974, 42.

95 Elizabeth Allen, ‘Baby Blues’, *The Stage* and *Television Today*, December 13, 1973, 21.

96 *Baby Blues*.

97 David Bradshaw, *Mirror*, January 11, 1996, 4.

98 Correspondence with Nemone Lethbridge, 12 May 2023.

99 *Baby Blues*.

100 Lethbridge, ‘Man on the Moon’, 88.

101 Michael Ratcliffe, ‘Play for Today BBC1’, *The Times*, December 7, 1973, 11.

102 British Library Oral Collection, C900:06552, 1998.

103 Mary Thomas, ed., *Post-War Mothers: Childbirth Letters to Grantly Dick-Read, 1946–1956* (Rochester, NY: University of Rochester Press, 1997).

104 *Baby Blues*.

105 Ibid.

106 Lethbridge, ‘Postscript to Baby Blues’, 17.

107 *Baby Blues*.

108 Val Arnold-Forster, ‘Trying to Couch It Another Way’, *Guardian*, October 2, 1982, 10.

109 Lethbridge, ‘Postscript to Baby Blues’, 17.

110 *Baby Blues*.

111 BBC WAC, *Woman’s Hour*, ‘Childbirth Depression’, Light Programme, May 17, 1960.

112 *Baby Blues*.

113 Correspondence with Nemone Lethbridge, 19 June 2023.

114 Margaret Allen, ‘Viewpoint’, *The Times*, August 13, 1973, 8; Kowk Hong Law, Ben Jackson, Kim Guelfi, et al. ‘Understanding and Alleviating Maternal Postpartum Distress: Perspectives from First-time Mothers’, *Social Science & Medicine* 204 (2019): 59–66.

115 Lethbridge, ‘Postscript to Baby Blues’, 17.

116 Barbara C. Stevens, ‘Psychoses Associated with Childbirth: A Demographic Survey since the Development of Community Care’, *Social Science & Medicine* 7, No.6 (1967): 52743.

117 Shaun Usher, ‘Mystery’, *Daily Mail*, December 7, 1973, 23.

118 Correspondence with Nemone Lethbridge, 12 May 2023.

119 Audience Research Reports; Office for National Statistics, ‘Population Estimates’, 21 December 2022 https://www.ons.gov.uk/peoplepopulationandcommunity/populationandmigration/populationestimates/timeseries/ukpop/pop (accessed February 22, 2024); May, ‘Play for Today’, 269.

120 James Glenn, ‘People Talk’, *Sunday People*, 13 January 1974, 8; Rowe, ‘Play For Today Shows Art Can Change Life’.

121 Rosemary Say, ‘Changing Gear’, *Sunday Telegraph*, December 9, 1973, 15.

122 D.A.N. Jones, ‘Forthcoming BBC Events’, *The Listener*, November 8, 1973, 650–1.

123 Russell Davis and John Bull, ‘Television’, *New Statesman*, December 17, 1973, 921.

124 Sean Day-Lewis, ‘Caricature Surrounds Hospital Play’, *Daily Telegraph*, December 7, 1972, 11.

125 Macmurraugh-Kavanagh, ‘Too Secret for Words’, 199.

126 Ratcliffe, ‘Play for Today BBC1’.

127 James Thomas, ‘Zena’s Birth Was Rather Hard to Bear’, *Daily Express*, December 7, 1973, 14.

128 Jack, ‘The Odd Couple’, 42; O’Connor, *The Eleventh Commandment*; Alana Harris and Timothy Willem Jones, eds., *Love and Romance in Britain, 1918–1970* (Basingstoke: Palgrave Macmillan, 2014).

129 Ratcliffe, ‘Play for Today BBC1’.

130 Davis and Bull, ‘Television’.

131 Ratcliffe, ‘Play for Today BBC1’.

132 Daniel Grey, ‘The Agony of Despair’: Pain and the Cultural Script of Infanticide in England and Wales, 1860–1960’, in *Pain and Emotion in Modern History*, ed. R. Boddice (Basingstoke: Palgrave Macmillan, 2014), 204–19.

133 Lethbridge, ‘Postscript to Baby Blues’, 17.

134 Thomas, ‘Zena’s Birth Was Rather Hard to Bear’; Ratcliffe, ‘Play for Today BBC1’.

135 BBC Written Archives, BBC/T/PRT/T14/1843/1; Salim Al-Gailani, ‘Drawing Aside the Curtain’: Natural Childbirth on Screen in 1950s Britain’, *British Society for the History of Science* 50 (2017): 473–93.

136 ‘Whitehouse on Television: The National Viewers’ and Listeners’ Association and Moral and Cultural Politics’, in Lawrence Black, *Redefining British Politics* (Basingstoke: Palgrave Macmillan, 2010), 105–138, 105–6.

137 Lethbridge, ‘Postscript to Baby Blues’, 17; Anon., ‘Mary gives TV ‘Baby Blues’ a Feast’, *Newcastle Evening Chronicle*, December 7, 1973, 11.

138 Jennings, Rebecca, ‘Lesbian Motherhood and the Artificial Insemination Scandal of 1978’, *Twentieth Century British History* 28 (2017): 570–94, 571–2; Mary Whitehouse, *Cleaning Up TV* (London, 1967), 23, cited in Martin Durham, *Sex and Politics: The Family and Morality in the Thatcher Years* (Basingstoke: Macmillan Education, 1991), 9.

139 Black, *Redefining British Politics*, 105–38.

140 Anon., ‘BBC Baby Play Has Mary Fuming’, *Daily Mail*, December 8, 1973, 8; Anon., ‘Mary Sees Red Over *Baby Blues*’, *Aberdeen Evening Express*, December 7, 1973, 11; Anon., ‘Mrs. Whitehouse Hits Out At ‘Birth’ Play’, *Belfast Telegraph*, December 7, 1973, 1; Lethbridge, ‘Postscript to Baby Blues’, 17.

141 Mrs R. Steed, ‘Letters. Oh, Baby’, *Daily Mail*, December 14, 1973, 25.

142 Lethbridge, ‘Postscript to Baby Blues’, 17.

143 BBC WAC, Board of Governors Meetings, ‘Baby Blues’, 1973/4, 21.

144 Lethbridge, ‘Postscript to Baby Blues’, 17.

145 Allen, ‘Baby Blues’, 21.

146 Usher, ‘Mystery’, 23.

147 BAC WAC, Audience Research Reports, *Baby Blues*.

148 Lethbridge, ‘Postscript to Baby Blues’, 17.

149 Jean Gardiner, ‘Depressives Anonymous’, *Spare Rib*, Issue 30, December 1974, 19–21.

150 Lethbridge, ‘Postscript to Baby Blues’, 17.

151 L. Stokes, ‘Letters. Baby Blues’, *Sunday Telegraph*, April 28, 1974, 11.

152 Lethbridge, ‘Not So Much Childbirth as Depression’, 76.

153 Anon., ‘Pregnancy’, *Women's Report* 2 (1974): 10.

154 Lethbridge, ‘Postscript to Baby Blues’, 17; Depressives Anonymous, ‘Self-Help’, 42.

155 Depressives Anonymous, ‘Self-Help’, 42.

156 Nemone Lethbridge, ‘First National Conference of Depressives Anonymous’, *Spare Rib*, Issue 28, October 1974, 32; Gardiner, ‘Depressives Anonymous’, 19–21.

157 Emma Ashworth, ‘An Interview with Jean Robinson’, Association for Improvements in the Maternity Services, 1 June 2018. https://www.aims.org.uk/journal/item/jean-robinson-aims-interview (accessed February 22, 2024).

158 Gardiner, ‘Depressives Anonymous’, 19–21; Lethbridge, ‘First National Conference of Depressives Anonymous’.

159 Rowe, ‘Play for Today Shows Art Can Change Life’.

160 Tim Lott, ‘Dorothy Rowe obituary’, *The Guardian*, April 11, 2019. https://www.theguardian.com/education/2019/apr/11/dorothy-rowe-obituary; Rowe, ‘Play for Today Shows Art Can Change Life’.

161 Gardiner, ‘Depressives Anonymous’.

162 Anne Gaines, ‘Odds & Sods: Depressives Anonymous’, *Spare Rib*, Issue 30, December 1974, 33.

163 Depression UK, ‘About Depression UK’, *Depression UK*. https://www.depressionuk.org/about-depressionuk/.

164 Anon., ‘The Sad, Sick, Sorry State’, *Guardian*, April 3, 1975, 11.

165 Vivienne Welburn, ‘Induction’, *Spare* Rib, Issue 38, August 1975, 8.

166 Sue Martin, ‘Projects: Postal Depressives Anonymous’, *Spare Rib*, Issue 63, October 1977, 25.

167 Vivienne Welburn, *Postnatal Depression* (Manchester: Manchester University Press, 1980), 9, 93.

168 ‘Nemone Lethbridge’, *The Light of Experience*.

169 Rowe, ‘Play for Today Shows Art Can Change Life’.

170 Alex Mold, *Making the Patient-Consumer: Patient Organisations and Health Consumerism in Britain* (Manchester: Manchester University Press, 2015), 22.

171 Depression UK, ‘About Depression UK’.

172 Sheila Kitzinger, ‘The Woman on the Delivery Table’, in Laing, *Woman on Woman*, 89–112; Correspondence with Nemone Lethbridge, 19 June 2023; Katharina Dalton, *Depression After Childbirth: How to Recognize and Treat Postnatal Illness* (New York, Oxford: Oxford University Press, 1980).

173 Welburn, *Postnatal Depression.*

